# Insights into the pathogenic character of a common *NUBPL* branch-site mutation associated with mitochondrial disease and complex I deficiency using a yeast model

**DOI:** 10.1242/dmm.012682

**Published:** 2013-07-04

**Authors:** Mateusz M. Wydro, Janneke Balk

**Affiliations:** 1 Department of Plant Sciences, University of Cambridge, Downing Street, Cambridge, CB2 3EA, UK; 2 Department of Biological Chemistry, John Innes Centre, Norwich, NR4 7UH, UK; 3 The School of Biological Sciences, University of East Anglia, Norwich, NR4 7TJ, UK

## Abstract

Complex I deficiencies are the most common causes of mitochondrial disorders. They can result from mutations not only in the structural subunits but also in a growing number of known assembly factors. A branch-site mutation in the human gene encoding assembly factor NUBPL has recently been associated with mitochondrial encephalopathy and complex I deficiency in seven independent cases. Moreover, the mutation is present in 1.2% of European haplotypes. To investigate its pathogenicity, we have reconstructed the altered C-terminus that results from the branch-site mutation and frameshift in the homologous Ind1 protein in the respiratory yeast *Yarrowia lipolytica*. We demonstrate that the altered sequence did not affect *IND1* mRNA stability, yet it led to a decrease in Ind1 protein level. The instability of mutant Ind1 resulted in a strong decrease in complex I activity and caused slow growth, resembling the phenotype of the deletion strain of *IND1*. The presented data confirms the deleterious impact of the altered C-terminus resulting from the branch-site mutation. Furthermore, our approach demonstrates the great potential of *Y. lipolytica* as a model to investigate complex I deficiencies, especially in cases with genetic complexity.

## INTRODUCTION

Mitochondrial complex I (also known as NADH:ubiquinone oxidoreductase, EC 1.6.5.3) plays a key role in oxidative phosphorylation (OXPHOS). Complex I couples the transfer of electrons generated in the tricarboxylic acid (TCA) cycle with proton translocation across the inner mitochondrial membrane. The proton pumping activity of complex I generates a significant fraction of proton motive force, which drives ATP synthesis. Complex I is one of the largest and the most complicated protein assemblies, with a molecular weight close to 1 MDa. In most eukaryotes, complex I consists of about 40+ subunits, from which the seven most hydrophobic are encoded in the mitochondrial genome (Hirst, 2013). The accurate assembly of this multi-protein complex involves the coordinated expression of two genomes and synchronized incorporation of a number of subunits and non-protein cofactors. Such a complicated multistage process requires assistance from a number of assembly factors. It has been estimated that complex I deficiency underlies between a quarter and a third of OXPHOS disorders (Bugiani et al., 2004; Loeffen et al., 2000; Scaglia et al., 2004; Thorburn, 2004), which overall are thought to affect ∼1 in 5000 births (Skladal et al., 2003). Until recent years, genetic diagnostics of complex I disorders based on sequencing of a known set of 44 genes encoding structural subunits of complex I (37 encoded in the nuclear genome and seven in the mitochondrial genome) could only offer a genetic explanation for about 50% of affected individuals (Calvo et al., 2010). This low proportion suggested the existence of many additional factors that are not integral to mature complex I, but are required for its effective assembly and function. The lack of complex I in baker’s yeast has held back identification of its assembly factors, yet the first two complex I assembly factors, named CIA30 and CIA84, were identified in another mitochondrial model organism, *Neurospora crassa* (Küffner et al., 1998). Since then the list of complex I assembly factors has steadily grown (recently reviewed in Nouws et al., 2012; Pagniez-Mammeri et al., 2012).

One of the complex I assembly factors, named Ind1, has been identified and characterised by our group (Bych et al., 2008), taking advantage of the yeast *Yarrowia lipolytica* as a model organism (Kerscher et al., 2002; Kerscher et al., 2004). Interestingly, it was noticed that the *IND1* gene is present, with only few exceptions, in the genomes of species that retain functional complex I (Bych et al., 2008). The *IND1* knockout in *Y. lipolytica* resulted in slower growth and a specific decrease in complex I activity. Owing to the fact that Ind1 is capable of binding a labile Fe-S cluster *in vitro* and displays sequence similarity to Nbp35 and Cfd1, scaffold proteins that are involved in cytosolic Fe-S cluster assembly, it was suggested that Ind1 plays a role in the assembly of one or more of the eight Fe-S clusters of complex I (Bych et al., 2008). The siRNA knockdown of the human homologue, *NUBPL* or *huIND1*, in HeLa cells led to a strong decrease in complex I activity and its subunit levels, as well as to an accumulation of aberrant assembly products in the form of subcomplexes (Sheftel et al., 2009). Based on the discovery of *IND1* in *Y. lipolytica*, the human homologue *NUBPL* was included in a list of 103 candidate genes for next-generation exon sequencing in a cohort of 103 patients with complex I deficiency (Calvo et al., 2010) (E. J. Tucker, Murdoch Childrens Research Institute, and University of Melbourne, Melbourne, Australia, personal communication). This high-throughput screen led to the identification of a missense mutation in exon 2 in the *NUBPL* gene (Calvo et al., 2010). The G to A substitution of nucleotide 166 (c.166G>A) results in substitution of glycine 56 to arginine (p.G56R). The patient was characterised as compound heterozygous for a complex gene rearrangement, including a deletion that spans exons 1–4 inherited on the maternal allele. As a consequence, the c.166G>A mutation on the paternal allele was apparent as homozygous. Further sequence analysis identified a second point mutation in intron 9 on the paternal allele, which is a branch-site mutation. The T to C substitution lies 27 nucleotides upstream from the 5′ end of exon 10 (c.815-27T>C) and results in aberrant splice products (Tucker et al., 2012). This branch-site mutation has recently been found in another six independent cases, usually in combination with a probable null allele (Tenisch et al., 2012; Kevelam et al., 2013). In most of these cases the c.166G>A mutation was also present on the same allele (Kevelam et al., 2013).

**TRANSLATIONAL IMPACT**

**Clinical issue**

Deficiency of respiratory complex I is the most frequent causative factor underlying mitochondrial disorders. Mitochondrial diseases are associated with a large diversity of clinical symptoms, among which encephalopathy is commonly observed. Disease-causing mutations have been found in the genes encoding complex I subunits or mitochondria-encoded tRNAs, as well as, recently, in several genes encoding complex I assembly factors. Within the latter group is the c.815-27T>C branch-site mutation, which affects splicing of the gene encoding the assembly factor NUBPL. This mutation was identified in seven unrelated individuals with mitochondrial disease; most of these are compound heterozygotes with one probable null allele, and the other allele carrying the branch-site mutation in combination with a c.166G>A missense mutation. A previous study reported that the missense mutation by itself does not cause detectable complex I deficiency; however, the experimental strategy involved overexpression of the *NUBPL* gene, which might have obscured any subtle defects. Owing to the complex genetics of the compound heterozygous patients, pathogenicity of the branch-site mutation, which occurs at a frequency of 1.2% in the European population, has not been verified thus far.

**Results**

In this work, the authors investigated the potential pathogenicity of the c.815-27T>C branch-site mutation using an established yeast model for studying complex I deficiency, *Yarrowia lipolytica*. They reconstructed the altered C-terminal coding sequence resulting from the branch-site mutation in the *Y. lipolytica* homologue of *NUBPL*, the *IND1* gene. The authors assessed the effects of mutant protein expression on assembly of complex I *in vivo*. The C-terminal mutation led to a severe decrease in the level of Ind1 protein, which had a deleterious impact on complex I activity, quantified as an 80% decrease in assembly and function. Mutant yeast demonstrated slow cell growth resembling the phenotype of the deletion strain of *IND1*. Expression of mutant *IND1* did not affect expression of the wild-type copy in heterozygotes, i.e. the mutation did not have a dominant-negative effect.

**Implications and future directions**

The data described here suggest that the branch-site mutation is involved in the pathophysiology of complex I deficiency. However, it is possible that the c.815-27T>C branch-site mutation is only pathogenic in combination with the c.166G>A missense mutation that is found in most of the reported patients. In this scenario, which remains to be tested, complex I deficiency could be explained by decreased expression levels of correctly spliced *NUBPL*, in combination with mildly compromised functionality due to the missense mutation. The results demonstrate the great potential of *Y. lipolytica* as a model to investigate mutations underlying complex I deficiencies, especially in cases with genetic complexity. Moreover, the reconstruction of mutations in this model system is rapid and technically straightforward. Despite the considerable evolutionary distance between human and yeast, analysis of patient mutations in *Y. lipolytica* has the potential to be translated to human complex I biology. Currently there is no cure for mitochondrial disease; however, unravelling the molecular mechanisms and genetics of complex I deficiency could enable improved diagnosis and treatment.

Interestingly, the transgene expression of mutant *NUBPL* (p.G56R) showed that the missense mutation does not impact substantially on *NUBPL* mRNA stability, protein stability, import in mitochondria, or its function (Tucker et al., 2012). Therefore, the c.815-27T>C branch-site mutation is most likely the cause of complex I deficiency, although an additive effect of the missense mutation cannot to be excluded. Reverse transcriptase PCR (RT-PCR) and sequence analysis showed that the branch-site mutation resulted in a profound decrease in the steady-state level of *NUBPL* mRNA, quantified as 15% of control levels. Two additional aberrant transcripts were observed, of which one was degraded by nonsense-mediated decay. The other aberrant transcript lacked exon 10. Exon 9 was directly fused to exon 11, causing a frameshift after glycine 272, with an additional 31 codons until a stop codon. As a consequence, aspartate 273 was replaced by a glutamine followed by 30 random amino acids, resulting in an altered C-terminus (p.D273QfsX31). The mutation leads to a decrease in NUBPL protein level (Tucker et al., 2012; Kevelam et al., 2013).

The presence of the c.815-27T>C mutation in several unrelated NUBPL patients should perhaps not be surprising in light of the fact that this branch-site mutation is found in 1.2% (7/751) of the European haplotypes (Tucker et al., 2012) (www.ensembl.org). It is therefore of high interest to determine the pathogenicity of the c.815-27T>C variant.

**Fig. 1.**
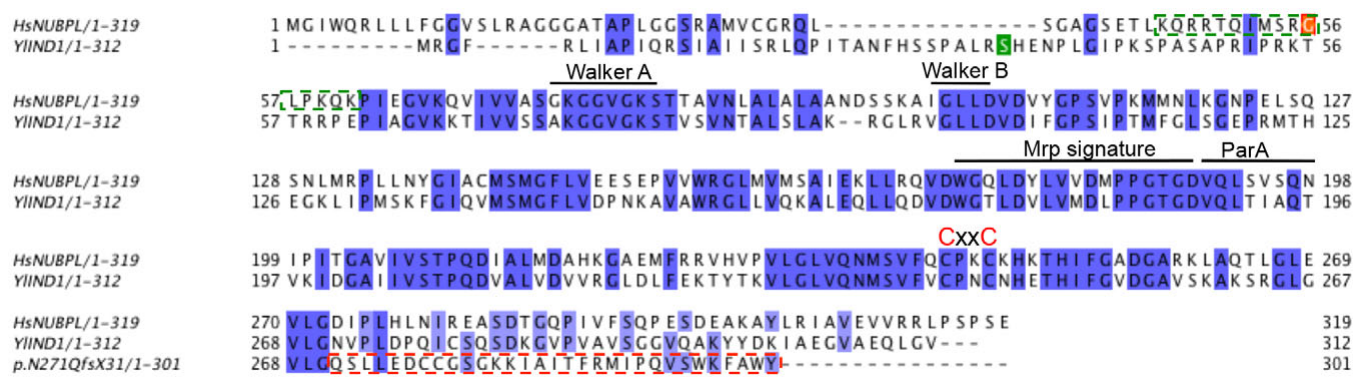
**Alignment of human NUBPL, *Yarrowia lipolytica* Ind1 and the frameshift mutant protein.** Clustal Omega alignment of human NUBPL (NP_079428.2), *Y. lipolytica* Ind1 (YALI0B18590p) and the 35 C-terminal amino acids of Ind1 p.N271QfsX31, of which the 31 C-terminal amino acids (boxed in red broken line) are based on the frameshift resulting from the c.815-27T>C mutation in *NUBPL*. The alignment was visualized using Jalview (Waterhouse et al., 2009). The conserved Walker A [GxxxxGK(T/S)] and Walker B (hhhhD) motifs (h denotes a hydrophobic residue), the Mrp signature [Wxx(L/I/V/M)D(V/F/Y)(L/I/V/M)3DxPPGT(G/S)D], the ParA domain, and the conserved CxxC (red) sequence are marked above the alignment. The experimentally verified mature N-terminus (Ser35) after cleavage of the mitochondrial targeting sequence in *Y. lipolytica* Ind1 (Bych et al., 2008) is marked green. In human NUBPL, Lys44 is thought to be the mature N-terminus, based on the KQRRTQIMSRGLPKQK peptide (boxed in green broken line) detected in the mouse mitochondrial proteome (Pagliarini et al., 2008). Gly56, affected by the c.166G>A mutation in the reported patients, is shown in orange.

We set out to investigate the impact of *NUBPL* c.815-27T>C on complex I assembly by recreating the altered C-terminus resulting from frameshift p.D273QfsX31 in the homologous Ind1 protein in *Y. lipolytica*. The G56 residue is not conserved in Ind1, and could therefore not be investigated (Fig. 1). *Y. lipolytica* is an obligate aerobic yeast has previously been used successfully as a model to reconstruct pathogenic human mutations in *NDUFS2*, *NDUFS3*, *NDUFS7* and *NDUFS8*, which encode structural subunits of complex I (Kerscher et al., 2004). In many cases, reconstruction of these mutations resulted in specific defects in complex I stability and function, correlating well with patient delivered data and providing a deeper insight into the molecular mechanism of these deleterious changes (Kerscher et al., 2004).

Here, we report that the altered C-terminus resulting from the frameshift causes a decrease in the *Y. lipolytica* Ind1 protein level and an 80% decrease in complex I assembly and function. The decrease in complex I activity very much resembles the deficiency caused by *IND1* complete knockout. The mutant does not affect the expression of a wild-type copy of *IND1* in a heterozygous situation.

## RESULTS AND DISCUSSION

### Ind1 protein with the human p.D273QfsX31 C-terminus is unable to complement the *Y. lipolytica ind1*Δ mutant

To investigate how the p.D273QfsX31 mutation affects the function of human NUBPL, we engineered the same C-terminal amino acid sequence in *Y. lipolytica* Ind1. The coding DNA sequence of the human p.D273QfsX31 C-terminus was codon-optimised for expression in *Y. lipolytica* and cloned in frame with codons 1–270 of *IND1* into plasmid pUB4 with the endogenous promoter of *IND1*. This change results in expression of the hybrid protein Ind1 p.N271QfsX31, with the frameshift introduced downstream of the conserved VLG motif (Fig. 1).

**Fig. 2.**
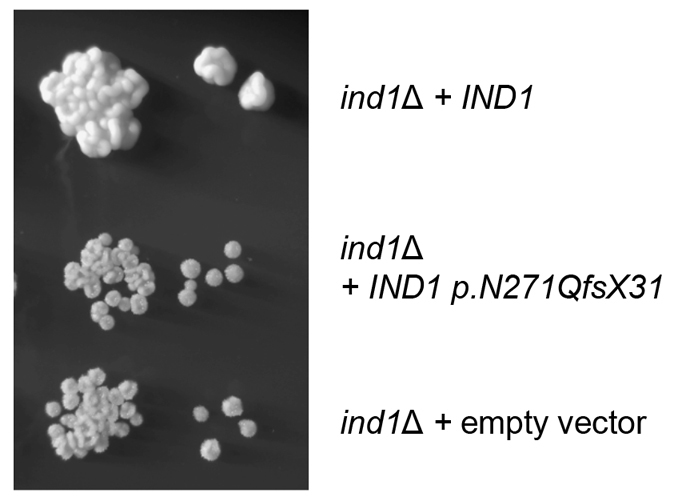
***IND1 p.N271QfsX31* under the control of the endogenous *IND1* promoter does not complement the *Y. lipolytica ind1*Δ mutant strain.** Overnight cultures of the complemented wild-type strain (*ind1*Δ *+ IND1*) and the *ind1*Δ strain expressing plasmid-borne *IND1 p.N271QfsX31* (*ind1*Δ *+ IND1 p.N271QfsX31*) or empty vector were grown in YPD + 50 μg/ml hygromycin B medium. The cultures were washed, and 5 μl of tenfold serial dilutions were spotted onto YPD plates containing 50 μg/ml hygromycin B, and incubated for 2 days at 28°C. The plates were stored for 5 days at 4°C before photographs were taken. The photograph of the two final serial dilutions is shown.

In order to determine the functional impact of Ind1 p.N271QfsX31, we performed complementation analysis. We used the haploid deletion mutant *ind1*Δ, obtained in *Y. lipolytica* by replacing the entire coding sequence of *IND1* with a *URA3* marker (Bych et al., 2008). The *ind1*Δ strain is able to bypass the absolute requirement for respiratory complex I owing to the expression of a transgenic version of the alternative NADH dehydrogenase (*NDH2i*) on the matrix side of the inner mitochondrial membrane (Kerscher et al., 2001). The *ind1*Δ strain is viable, but displays significantly slower growth. The growth of the *ind1*Δ strain could be fully restored following complementation with a plasmid expressing the full-length *IND1* gene, under the control of its endogenous promoter (Fig. 2, top). In contrast, the strain expressing *IND1 p.N271QfsX31* (middle) formed small and smooth colonies, resembling those of the *ind1*Δ strain transformed with the empty vector (bottom). These findings indicate that expression of *IND1 p.N271QfsX31* is unable to rescue the slow growth of *ind1*Δ.

### IND1 p.N271QfsX31 does not affect its mRNA but decreases protein stability

The observed incapability of Ind1 p.N271QfsX31 to rescue the growth phenotype of *ind1*Δ could be explained by four possibilities: (i) the introduced *p.N271QfsX31* coding sequence could potentially trigger an mRNA surveillance mechanism, leading to transcript degradation (nonsense-mediated decay); (ii) introduced changes in the protein structure could lead to misfolding and instability or; (iii) loss of function and, finally; (iv) expression of the *IND1 p.N271QfsX31* could lead to deleterious gain of function or a dominant negative activity. The latter possibility was experimentally excluded by testing the expression of a plasmid-borne *IND1 p.N271QfsX31* in the heterozygous strain *ind1*Δ*/+*. However, we did not notice any negative effect of expression of *IND1 p.N271QfsX31* on the growth of *ind1*Δ*/+* (data not shown).

**Fig. 3.**
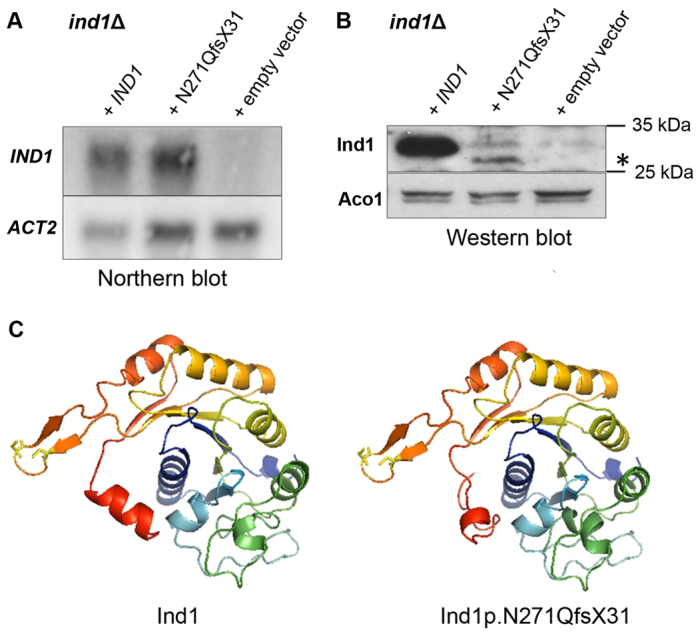
**Ind1 p.N271QfsX31 protein has decreased stability.** (A) Steady-state transcript levels of *IND1* or *IND1 p.N271QfsX31.* 10 μg of total RNA was analysed by northern blot using PCR-generated probes against *IND1* and cytosolic actin (*ACT2*) as a loading control. (B) Polyclonal antisera raised against recombinant Ind1 recognised the mature mitochondrial form of Ind1 (29.36 kDa) in the complemented wild-type strain (*ind1*Δ *+ IND1*) and a truncated form (marked as *) in the *ind1*Δ strain expressing plasmid-borne *IND1 p.N271QfsX31* (*ind1*Δ *+ IND1 p.N271QfsX31*). 50 μg of protein cell extract was loaded per lane. Polyclonal antibody against mitochondrial aconitase (Aco1) was used as a loading control. (C) 3D models of Ind1 and Ind1 p.N271QfsX31 generated by the Phyre2 server. The models are based on the crystal structure of nucleotide-binding protein af2382 from *Archaeoglobus fulgidus* (c2ph1A), which has 42% sequence identity. The N-terminal mitochondrial targeting sequence was not modelled. The conserved Cys-xx-Cys (CxxC) motif protrudes from the structure (Cys residues indicated in yellow). In the mutant protein (Ind1 p.N271QfsX31) the C-terminal 47 amino acids are replaced by a 31 amino acid sequence with no sequence homology. As a result, the C-terminal alpha helix (red) is shortened, and presumably its interaction with alpha helices a2 (cyan) and a1 (blue) are altered.

Next, we investigated the steady-state level of *IND1* transcripts in *Y. lipolytica*. Northern blot analysis of RNA isolated from the *ind1*Δ strain expressing *IND1 p.N271QfsX31* revealed no significant change in steady-state level of the transcript compared with the complemented wild-type (*ind1*Δ *+ IND1*) (Fig. 3A). *IND1* transcript could not be detected in the *ind1*Δ strain transformed with an empty vector (Fig. 3A).

The level of Ind1 p.N271QfsX31 protein was examined by western blotting, using a polyclonal antibody against Ind1. A weak immuno-signal of lower apparent molecular mass than wild-type Ind1 was detected specifically in the *ind1*Δ strain expressing *IND1 p.N271QfsX31* (Fig. 3B). The observed change in migration corresponded to the lower predicted molecular mass of Ind1 p.N271QfsX31 after maturation upon mitochondrial import (predicted 28.63 kDa). Despite the fact that both constructs were expressed from pUB4 vector and under control of the same *IND1* endogenous promoter, the truncated protein was present at a severely decreased level compared with wild-type Ind1 expression (*ind1*Δ *+ IND1*). This result indicates that the altered C-terminus led to protein instability, in agreement with decreased NUBPL levels reported in two unrelated cases (Tucker et al., 2012; Kevelam et al., 2013).

To assess the theoretical impact of p.N271QfsX31 on Ind1 folding, we modelled the secondary structure of Ind1 p.N271QfsX31 and wild-type Ind1. The introduction of N271Q and the following sequence of 30 random amino acids results in a model with a much shorter C-terminal alpha helix, and probably affects its interaction with alpha helices a2 and a1 (Fig. 3C). Similar changes in secondary structure were noticed in 3D models generated for NUBPL and NUBPL p.D273QfsX31 (data not shown).

### Ind1 p.N271QfsX31 cannot support complex I assembly

**Fig. 4.**
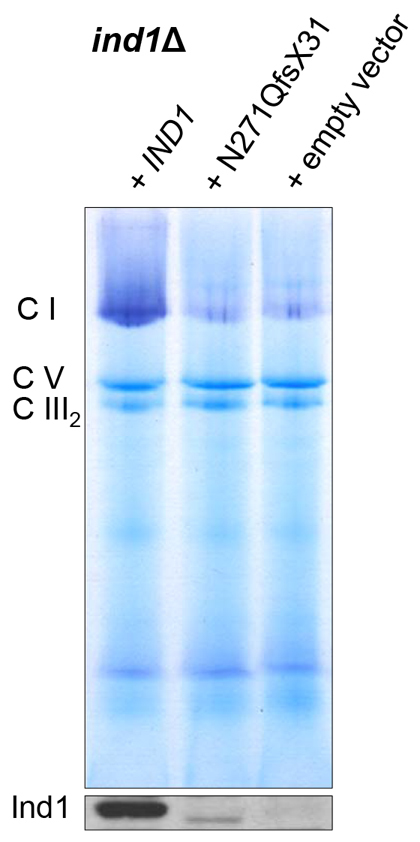
**Expression of Ind1 p.N271QfsX31 fails to restore complex I activity.** Mitochondrial membrane proteins of the strains as shown in Fig. 3 were separated by BN-PAGE and complex I activity was visualised by in NADH/NBT gel-staining. Monomeric complex I (with a molecular mass of 947 kDa) is marked as CI, F_o_F_1_ ATP synthase as CV and the dimer of complex III (cytochrome *bc*_1_ oxidoreductase) as CIII_2_. Immunoblot analysis of Ind1 protein levels in the corresponding samples is shown in the bottom panel.

We previously showed that slow growth of the *IND1* knockout strain is caused by complex I deficiency (Bych et al., 2008). To investigate the impact of Ind1 p.N271QfsX31 on complex I, we isolated unsealed mitochondrial membranes and separated the membrane complexes using blue-native PAGE (BN-PAGE). Complex I activity was then visualised using NADH and nitroblue tetrazolium (NBT). The intensity of the major activity associated with monomeric, fully assembled complex I (Fig. 4, CI) was strongly decreased in membranes from both *ind1*Δ yeast and *ind1*Δ expressing *IND1 p.N271QfsX31*, compared with the complemented wild type (*ind1*Δ *+ IND1*). Approximately 20% of intact, active complex I was previously reported to be present in the *ind1*Δ mutant (Bych et al., 2008). No significant differences were found between *ind1*Δ and the complemented wild-type strain for the other respiratory chain complexes, including succinate dehydrogenase (complex II); cytochrome *bc*_1_ complex (complex III) and ATP synthase (complex V) (Fig. 4 and data not shown). In *NUBPL* patients, complex I levels were reported to range between 27 and 83%, whereas the other respiratory chain complexes showed normal activities (Calvo et al., 2010; Tenisch et al., 2012; Kevelam et al., 2013).

In conclusion, the data presented here confirm the deleterious character of the altered C-terminus resulting from the c.815-27T>C branch-site mutation. The equivalent mutant *Y. lipolytica* protein (Ind1 p.N271QfsX31) is completely non-functional; therefore, in the patients reported so far, residual complex I activity (>20%) is likely to be a product of the small population (15%) of transcript that is processed correctly despite the branch-site mutation (Tucker et al., 2012).

Our approach further demonstrates the great potential of *Y. lipolytica* as a model to investigate complex I deficiencies not only in genes encoding structural proteins (Kerscher et al., 2004), but also in assembly factors (this study). Furthermore, the model is useful in unravelling the genetic complexity of disease-associated alleles.

## MATERIALS AND METHODS

### Yeast strains and constructs

The haploid *Y. lipolytica IND1* deletion strain *ind1*Δ has been described previously (Bych et al., 2008). In brief, the *IND1* gene (systematic name YALI0B18590g, GeneID: 2907193) open reading frame plus 27 bp upstream and 60 bp downstream were replaced by the *URA3* marker gene in the haploid GB10 strain (*ura3-302*, *leu2-270*, *lys11-23*, *NUGM-Htg2*, *NDH2i*, *MatB*) using homologous recombination. A plasmid-borne copy of *IND1* was generated by cloning the entire *IND1* gene, including the 1329 nucleotides upstream of the start codon that contains the endogenous promoter, into the pUB4 plasmid (Bych et al., 2008). The 1142-bp fragment of *IND1 p.N271QfsX31* was synthesised using *Y. lipolytica* codon optimization (GenScript, USA) and subcloned in-frame between *NsiI-EcoRI* to pUB4-*IND1*, resulting in pUB4-*IND1-p.N271QfsX31*. The coding sequence and cloning sites were confirmed by sequencing.

*Y. lipolytica* cells were routinely grown at 28°C in rich medium containing 1% (w/v) glucose (YPD). Hygromycin B (50 μg/ml) was added for the selection of cells transformed with the pUB4 plasmid.

### Blue native gel electrophoresis

Isolation of unsealed mitochondrial membranes was performed as described previously (Kerscher et al., 1999). Separation of the respiratory chain components that are present in mitochondrial membranes of *Y. lipolytica* was carried out using BN-PAGE essentially as previously described (Wittig et al., 2006), with some minor modifications. Unsealed membranes (200 μg of protein) were solubilised with dodecyl maltoside (2 g/g of protein) and 500 mM amino caproic acid and separated on non-denaturing 4–16% NativePAGE™ Novex Bis-Tris gels (Invitrogen). For in-gel detection of the NADH dehydrogenase activity of complex I, BN-PAGE gels were incubated for 10–15 minutes in a solution containing 3 mM NBT and 120 mM NADH. To stop the reaction, gels were incubated in 50% (v/v) methanol and 10% (v/v) acetic acid.

### Western blot analysis

Total cell lysates were prepared by vortexing cells with glass beads in lysis buffer [100 mM Tris-Cl pH 8.0, 150 mM NaCl, 1 mM EDTA, 5% (v/v) glycerol, 0.5% (v/v) Triton X-100, containing 2 mM PMSF, 15×1 minute vortex, 1 minute on ice]. 50 μg of protein extract (normalized by Bradford assay) was analysed by western blotting using a rabbit polyclonal antibody raised against purified Ind1 (Bych et al., 2008) and antisera against Aco1p (Kispal et al., 1999).

### RNA isolation and northern blot analysis

0.2 g of cells harvested from overnight YPD cultures were washed in distilled water and resuspended in 2 ml TRIzol reagent (Invitrogen). The cells were broken with 1 g glass beads (0.45–0.5 mm) in three cycles of 1 minute vortexing followed by 1 minute incubation on ice. RNA was then extracted following the manufacturer’s recommendations. RNA samples (10 μg) were denatured and separated through a 1.2% agarose, 1% (w/v) formaldehyde gel (3 hours at 60 V in 0.04 M MOPS, 10 mM sodium acetate, 1 mM EDTA pH 8.0) and transferred by capillary blotting overnight to HybondN+ membrane (GE Healthcare Life Sciences). Membranes were prehybridized for 1 hour in ULTRAhyb buffer (Ambion) before addition of radiolabelled probes. The probes were generated by PCR with use of the following primers pairs: ACT2-F 5′-TATGTGCAAGGCCGGTTTCG-3′, ACT2-R 5′-TCGATGGGGTATCGGAGGGT-3′ and IND1-F 5′-CGTCTCCGTGAACACAGCAC-3′, IND1-R 5′-CACGCCACTGCCTTGTTAG-3′. The PCR products were ^32^P-labelled with Rediprime II DNA Labeling System™ (GE Healthcare Life Sciences). Blots were incubated with the probes at 42°C overnight, rinsed, and washed twice in 2× SSC, 0.5% (w/v) SDS for 30 minutes at 42°C. Northern blots were visualized by PhosphorImager analysis (GE Healthcare Life Sciences). Blots were stripped in 0.1× SSC, 0.1% (w/v) SDS at 80°C for 30 minutes.

## References

[b1-0061279] Bugiani M. Invernizzi F. Alberio S. Briem E. Lamantea E. Carrara F. Moroni I. Farina L. Spada M. Donati M. A. (2004). Clinical and molecular findings in children with complex I deficiency. Biochim. Biophys. Acta 1659, 136–147 15576045 10.1016/j.bbabio.2004.09.006

[b2-0061279] Bych K. Kerscher S. Netz D. J. Pierik A. J. Zwicker K. Huynen M. A. Lill R. Brandt U. Balk J. (2008). The iron-sulphur protein Ind1 is required for effective complex I assembly. EMBO J. 27, 1736–1746 18497740 10.1038/emboj.2008.98 PMC2435133

[b3-0061279] Calvo S. E. Tucker E. J. Compton A. G. Kirby D. M. Crawford G. Burtt N. P. Rivas M. Guiducci C. Bruno D. L. Goldberger O. A. (2010). High-throughput, pooled sequencing identifies mutations in *NUBPL* and *FOXRED1* in human complex I deficiency. Nat. Genet. 42, 851–858 20818383 10.1038/ng.659 PMC2977978

[b4-0061279] Hirst J. (2013). Mitochondrial complex I. Annu. Rev. Biochem. 82, 551–575 23527692 10.1146/annurev-biochem-070511-103700

[b5-0061279] Kerscher S. J. Okun J. G. Brandt U. (1999). A single external enzyme confers alternative NADH:ubiquinone oxidoreductase activity in Yarrowia lipolytica. J. Cell Sci. 112, 2347–2354 10381390 10.1242/jcs.112.14.2347

[b6-0061279] Kerscher S. J. Eschemann A. Okun P. M. Brandt U. (2001). External alternative NADH:ubiquinone oxidoreductase redirected to the internal face of the mitochondrial inner membrane rescues complex I deficiency in Yarrowia lipolytica. J. Cell Sci. 114, 3915–3921 11719558 10.1242/jcs.114.21.3915

[b7-0061279] Kerscher S. Dröse S. Zwicker K. Zickermann V. Brandt U. (2002). Yarrowia lipolytica, a yeast genetic system to study mitochondrial complex I. Biochim. Biophys. Acta 1555, 83–91 12206896 10.1016/s0005-2728(02)00259-1

[b8-0061279] Kerscher S. Grgic L. Garofano A. Brandt U. (2004). Application of the yeast Yarrowia lipolytica as a model to analyse human pathogenic mutations in mitochondrial complex I (NADH:ubiquinone oxidoreductase). Biochim. Biophys. Acta 1659, 197–205 15576052 10.1016/j.bbabio.2004.07.006

[b9-0061279] Kevelam S. H. Rodenburg R. J. Wolf N. I. Ferreira P. Lunsing R. J. Nijtmans L. G. Mitchell A. Arroyo H. A. Rating D. Vanderver A. (2013). NUBPL mutations in patients with complex I deficiency and a distinct MRI pattern. Neurology 80, 1577–1583 23553477 10.1212/WNL.0b013e31828f1914 PMC3662327

[b10-0061279] Kispal G. Csere P. Prohl C. Lill R. (1999). The mitochondrial proteins Atm1p and Nfs1p are essential for biogenesis of cytosolic Fe/S proteins. EMBO J. 18, 3981–3989 10406803 10.1093/emboj/18.14.3981 PMC1171474

[b11-0061279] Küffner R. Rohr A. Schmiede A. Krüll C. Schulte U. (1998). Involvement of two novel chaperones in the assembly of mitochondrial NADH:Ubiquinone oxidoreductase (complex I). J. Mol. Biol. 283, 409–417 9769214 10.1006/jmbi.1998.2114

[b12-0061279] Loeffen J. L. Smeitink J. A. Trijbels J. M. Janssen A. J. Triepels R. H. Sengers R. C. van den Heuvel L. P. (2000). Isolated complex I deficiency in children: clinical, biochemical and genetic aspects. Hum. Mutat. 15, 123–134 10649489 10.1002/(SICI)1098-1004(200002)15:2<123::AID-HUMU1>3.0.CO;2-P

[b13-0061279] Nouws J. Nijtmans L. G. J. Smeitink J. A. Vogel R. O. (2012). Assembly factors as a new class of disease genes for mitochondrial complex I deficiency: cause, pathology and treatment options. Brain 135, 12–22 22036961 10.1093/brain/awr261

[b14-0061279] Pagliarini D. J. Calvo S. E. Chang B. Sheth S. A. Vafai S. B. Ong S. E. Walford G. A. Sugiana C. Boneh A. Chen W. K. (2008). A mitochondrial protein compendium elucidates complex I disease biology. Cell 134, 112–123 18614015 10.1016/j.cell.2008.06.016 PMC2778844

[b15-0061279] Pagniez-Mammeri H. Loublier S. Legrand A. Bénit P. Rustin P. Slama A. (2012). Mitochondrial complex I deficiency of nuclear origin I. Structural genes. Mol. Genet. Metab. 105, 163–172 22142868 10.1016/j.ymgme.2011.11.188

[b16-0061279] Scaglia F. Towbin J. A. Craigen W. J. Belmont J. W. Smith E. O. Neish S. R. Ware S. M. Hunter J. V. Fernbach S. D. Vladutiu G. D. (2004). Clinical spectrum, morbidity, and mortality in 113 pediatric patients with mitochondrial disease. Pediatrics 114, 925–931 15466086 10.1542/peds.2004-0718

[b17-0061279] Sheftel A. D. Stehling O. Pierik A. J. Netz D. J. Kerscher S. Elsässer H. P. Wittig I. Balk J. Brandt U. Lill R. (2009). Human ind1, an iron-sulfur cluster assembly factor for respiratory complex I. Mol. Cell. Biol. 29, 6059–6073 19752196 10.1128/MCB.00817-09 PMC2772561

[b18-0061279] Skladal D. Halliday J. Thorburn D. R. (2003). Minimum birth prevalence of mitochondrial respiratory chain disorders in children. Brain 126, 1905–1912 12805096 10.1093/brain/awg170

[b19-0061279] Tenisch E. V. Lebre A.-S. Grévent D. de Lonlay P. Rio M. Zilbovicius M. Funalot B. Desguerre I. Brunelle F. Rötig A. (2012). Massive and exclusive pontocerebellar damage in mitochondrial disease and *NUBPL* mutations. Neurology 79, 391 22826544 10.1212/WNL.0b013e3182611232

[b20-0061279] Thorburn D. R. (2004). Mitochondrial disorders: prevalence, myths and advances. J. Inherit. Metab. Dis. 27, 349–362 15190193 10.1023/B:BOLI.0000031098.41409.55

[b21-0061279] Tucker E. J. Mimaki M. Compton A. G. McKenzie M. Ryan M. T. Thorburn D. R. (2012). Next-generation sequencing in molecular diagnosis: *NUBPL* mutations highlight the challenges of variant detection and interpretation. Hum. Mutat. 33, 411–418 22072591 10.1002/humu.21654

[b22-0061279] Waterhouse A. M. Procter J. B. Martin D. M. A. Clamp M. Barton G. J. (2009). Jalview Version 2 – a multiple sequence alignment editor and analysis workbench. Bioinformatics 25, 1189–1191 19151095 10.1093/bioinformatics/btp033 PMC2672624

[b23-0061279] Wittig I. Braun H.-P. Schägger H. (2006). Blue native PAGE. Nat. Protoc. 1, 418–428 17406264 10.1038/nprot.2006.62

